# Evaluation of tonsillectomy in the treatment of SAPHO syndrome: past evidence and future directions

**DOI:** 10.3389/fmed.2025.1695572

**Published:** 2025-12-10

**Authors:** Wen-Yuan Gan, Ben-Hong Ren, Qing-Ping Zhang, Yi Wang

**Affiliations:** 1 Qinghai University Affiliated Hospital, Xining, Qinghai, China; 2 Department of Otolaryngology, Peking Union Medical College Hospital, Chinese Academy of Medical Sciences and Peking Union Medical College, Beijing, China

**Keywords:** SAPHO syndrome, treatment, tonsillectomy, palmoplantar pustulosis, synovitis, acne, hyperostosis, osteomyelitis

## Abstract

Synovitis, acne, pustulosis, hyperostosis, and osteitis (SAPHO) syndrome is a rare aseptic inflammatory clinical syndrome characterized by widespread skin lesions and bone-joint lesions. The underlying mechanisms remain unclear, making it difficult to treat. Currently, drug-based symptomatic treatment is the main approach, but symptoms often recur or worsen when medications are reduced or discontinued. Recently, there have been increasing reports of the significant therapeutic effects of tonsillectomy in treating SAPHO syndrome. This paper reviews the past evidence on the use of tonsillectomy for SAPHO syndrome by searching PubMed, Google Scholar, Web of Science, Embase, Scopus, CNKI, and Wanfang databases using the keywords “SAPHO syndrome and tonsillectomy.” It also discusses the potential mechanisms of tonsillectomy in treating SAPHO syndrome, aiming to provide new treatment methods for SAPHO syndrome.

## 1 Introduction

SAPHO syndrome (which stands for synovitis, acne, pustulosis, hyperostosis, and osteitis) is a chronic, recurrent, aseptic inflammatory disease first reported by French rheumatologist Chamot in 1986 (1). The primary symptoms include synovitis, acne, pustulosis, hyperostosis, and osteitis (2). Its clinical manifestations, pathogenesis, and treatment have been the focus of researchers. The incidence rate of SAPHO syndrome in Caucasians is approximately 1 per 100,000 (2, 3). Due to its rarity, the interval between skin and bone symptoms can span several years, leading to misdiagnosis and underestimation of the incidence rate (4).

The exact mechanism of SAPHO syndrome remains unclear, but current evidence suggests that it is caused by a combination of immune dysregulation, genetic susceptibility, and environmental factors (5–7). To date, there is no unified treatment protocol, and drug-based symptomatic treatment is the primary method. Treatment drugs are generally divided into three lines: First-line drugs primarily consist of non-steroidal anti-inflammatory drugs (NSAIDs) such as ibuprofen, naproxen, indomethacin, and diclofenac, which are used to alleviate inflammation, pain, and discomfort in bones and joints. Corticosteroids are also used as first-line drugs, especially during acute episodes. They help reduce inflammation and alleviate joint, skin, and bone symptoms. For patients who do not respond well to NSAIDs or corticosteroids, second-line drugs, including disease-modifying antirheumatic drugs (DMARDs), are often required. Traditional DMARDs like methotrexate, sulfasalazine, cyclosporine A, cyclophosphamide, and thalidomide suppress the overactive immune system to slow disease progression. Biologic DMARDs can be another treatment option, particularly when traditional treatments (such as NSAIDs and corticosteroids) are ineffective. Bisphosphonates are commonly used as second-line drugs, especially in patients with significant bone symptoms or damage. Bisphosphonates mainly alleviate bone pain and improve bone symptoms by inhibiting bone resorption, making them particularly effective for patients with bone inflammation or osteoporosis. In some cases where bisphosphonates are ineffective, they can be used as adjunctive therapy. Tumor necrosis factor inhibitors (TNF inhibitors), such as infliximab, adalimumab, and etanercept, are biologic DMARDs that reduce inflammation and bone damage by inhibiting TNF, making them the preferred treatment for many rheumatic diseases, including SAPHO syndrome. Additionally, IL-1 inhibitors and IL-6 inhibitors regulate inflammatory responses in the immune system. Recent studies have shown JAK inhibitors effectiveness in treating SAPHO syndrome, particularly for patients unresponsive to traditional drugs (8–12). JAK inhibitors regulate the immune system by inhibiting the JAK-STAT signaling pathway, thereby reducing inflammation (13, 14). Third-line drugs are typically used when first- and second-line treatments are ineffective. These drugs are aimed at alleviating symptoms and slowing disease progression, especially in refractory cases. Common third-line drugs include antibiotics (tetracyclines), immunosuppressants (cyclophosphamide), and other inhibitors such as B cell depleting agents like rituximab. Cheng et al. (15) summarized treatment strategies for SAPHO syndrome, which are usually adjusted based on the patient’s clinical presentation, disease severity, and drug response, to develop individualized treatment plans. Given the complex etiology of the disease, both first- and second-line drugs are symptomatic treatments that cannot cure the disease fundamentally. Long-term medication side effects and symptom recurrence upon dose reduction or discontinuation are unavoidable.

Palmoplantar pustulosis (PPP) is one of the symptoms of SAPHO syndrome, and pustulotic arthro-osteitis (POA) is a joint complication of PPP. Tonsillectomy has been proven effective in treating PPP and POA (16). Recent studies suggest that tonsillectomy can effectively improve skin symptoms like PPP and bone-joint symptoms in SAPHO syndrome (17). Wang et al. (18) reported that about two-thirds of patients had chronic tonsillitis, and after undergoing tonsillectomy, the patients’ skin and musculoskeletal symptoms significantly improved or disappeared. This paper retrospectively summarizes the treatment outcomes of tonsillectomy in SAPHO syndrome patients with chronic tonsillitis, hoping to provide a new treatment method for SAPHO syndrome.

## 2 Methods

**FIGURE 1:**
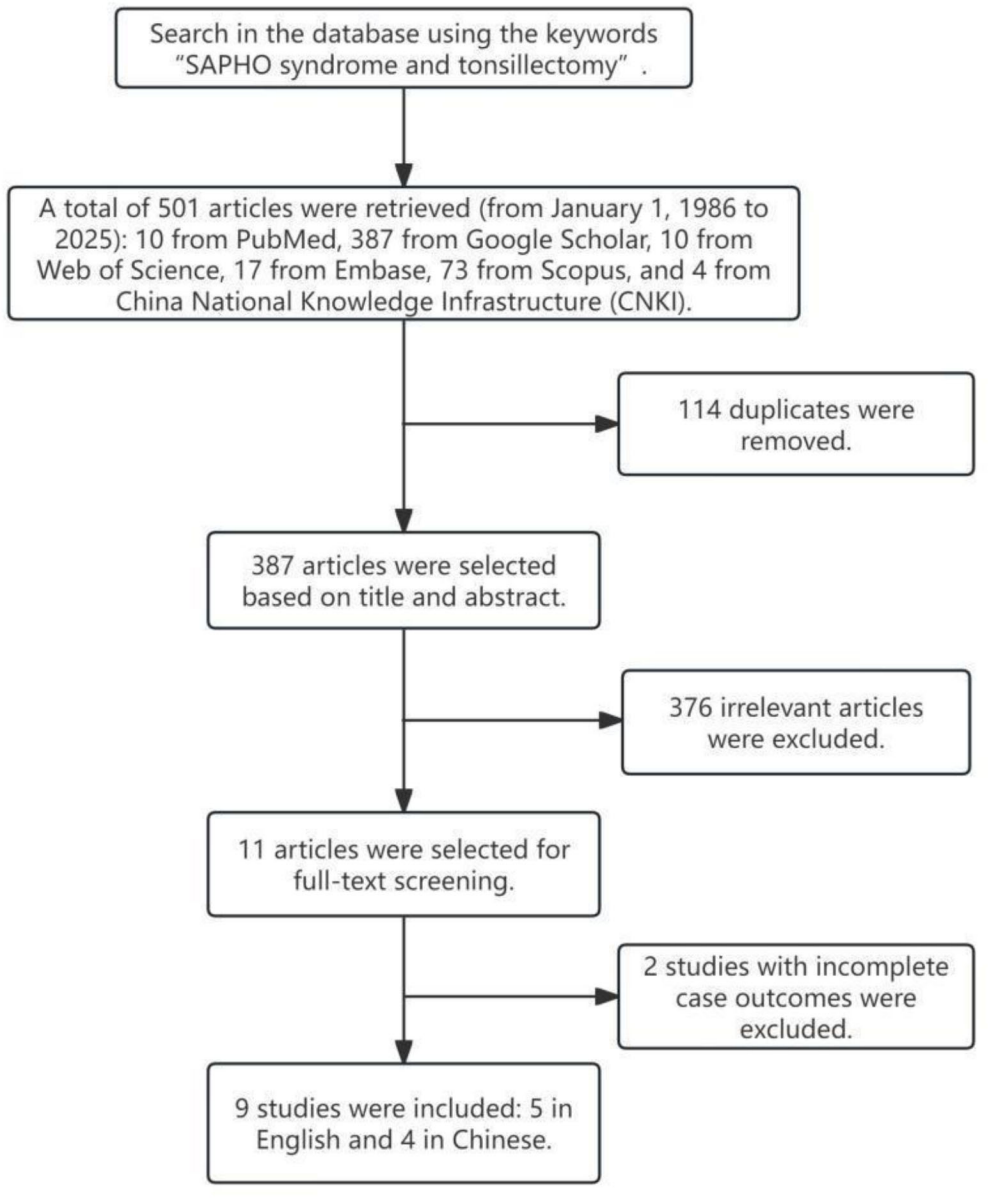
Search strategy for tonsillectomy treatment in synovitis, acne, pustulosis, hyperostosis, and osteitis (SAPHO) syndrome.

We searched PubMed, Google Scholar, Web of Science, Embase, Scopus, CNKI, and Wanfang databases using the keywords “SAPHO syndrome and tonsillectomy” for relevant English and Chinese literature. Inclusion criteria were case reports, case-control studies, and retrospective cohort studies with complete preoperative and postoperative records of tonsillectomy. Exclusion criteria included lack of complete follow-up data and follow-up time less than 1 month (Figure 1). We summarized the clinical manifestations (skin, bone-joint, other areas), tonsillitis status, and post-tonsillectomy efficacy of these SAPHO patients.

## 3 Results

Nine articles were included, all case reports, involving 58 patients from Asia, with 3 cases (5%) from Japan and 55 cases (94%) from China.

The clinical manifestations (skin, bone-joint, other areas), tonsillitis status, and posttonsillectomy efficacy of 58 SAPHO patients are summarized in Table 1. These patients, in addition to having palmoplantar pustulosis and joint pain, also suffered from varying degrees of chronic tonsillitis. One Japanese female patient also had hypertrophic meningitis (19). Another patient experienced irreversible hearing loss due to affected ossicles (20). One patient developed gangrene on the left lower leg (21). Before surgery, almost all patients had used first-line NSAIDs, second-line DMARDs, or a combination of both to control their condition. Overall, drug therapy could temporarily relieve most patients’ symptoms, but symptoms worsened or recurred after dose reduction or discontinuation. After tonsillectomy, 25% (15/58) of patients experienced worsening symptoms. One patient’s PPP worsened one week post-surgery (22), and another patient experienced irreversible hearing loss (20). Approximately one month post-surgery, eight patients experienced worsening rashes, and five patients felt more severe bone pain (18).

**TABLE 1:** The effectiveness of tonsillectomy in the treatment of synovitis, acne, pustulosis, hyperostosis, and osteitis (SAPHO) syndrome.

References	Number Of patients	Osteoarticular symptoms	Cutaneous symptoms	Other symptoms	Preperative treatment	After treatment	Follow-up visittinme	Adverse effects (AE)
1. Shimizu et al. (20)	1	1/1	1/1	Right ear Fluctuating hearing loss	Corticosteroids, Cyclosporine, Immunosuppressants	Ossicular Reconstruction, Cyclosporine, Methotrexate	1 week–7 months	Irreversible hearing loss
2. Shiraishi et al. (19)	1	1/1	1/1	Hypertrophic meningitis, eosinophilia	Methylprednisolone (1000 mg/day for 3 days), Prednisolone (10 mg/day), Minocycline (100 mg/day)	Prednisone	1week–10 months	NO
3. Horiguchi et al. (23)	1	1/1	1/1	NO	Celecoxib, Methotrexate, Adalimumab	Celecoxib, Methotrexate	3 months–12 months	NO
4. Wang et al. (18)	44	44/44	44/44	NO	NSAIDs, Glucocorticoids, DMARDs, Antibiotics, Traditional Chinese Medicine (TCM), Bisphosphonates, TNF-α Receptor Antagonists	NO	1 months–48 months	1 month after surgery:8/44 PPP worsened compared to before the surgery. 5/44 Bone pain worsened compared to before the surgery. Subsequently, both the rash and bone pain gradually eased.
5. Jia et al. (44)	1	1/1	1/1	NO	NO	NO	5 month-18 m onths	NO
6. Xie et al. (45)	1	1/1	1/1	NO	Minocycline Capsules, Ciclopirox, Halometasone Ointment, Celecoxib Capsules	NO	1 month	NO
7. Xiang et al. (17)	7	7/7	7/7	NO	NSAIDs (Etoricoxib), DMARDs (Etanercept), Methotrexate, Leflunomide)	2/7 Etanercept	3 weeks–2 months	NO
8. Ma et al. (21)	1	1/1	1/1	Left leg gangrene, nasosinusitis	Bisphosphonates, Glucocorticoids	NO	12 mnths	NO
9. Wang et al. (22)	1	1/1	1/1	NO	NSAIDs, Minocycline, Tripterygium Wilfordii	NO	1 week–48 months	Postoperatively, her palmar and digital pustules worsened, but the good news is that they improved after 2 weeks, and by 6 months, the PPP had completely disappeared. Two years later, the nasopharyngeal symptoms disappeared.

About 89% (52/58) of patients did not require drug therapy post-surgery, and about 96% (56/58) showed sustained symptom relief 1 month post-surgery, with three patients followed up for 2 years showing 100% resolution of skin and bone lesions (18). Only about 8% (5/58) of patients needed continued drug therapy post-surgery, with reduced medication doses compared to pre-surgery, gradually decreasing over time (23). One patient began reducing methotrexate dosage 3 months post-surgery and no longer required celecoxib and methotrexate six months post-surgery (23).

## 4 Tonsillectomy for SAPHO syndrome

Synovitis, acne, pustulosis, hyperostosis, and osteitis (SAPHO) syndrome presents with heterogeneous clinical manifestations, and not all patients experience concurrent skin and bone-joint symptoms. The disease follows a relapsing-remitting course, with varying severity, frequency, and location of lesions. Skin lesions are the most common symptoms in SAPHO syndrome, affecting 60%–90% of patients (24), with palmoplantar pustulosis (PPP), psoriasis vulgaris (PV), and severe acne (SA) being the most prevalent (24, 25). Skin involvement is often more severe than bone-joint involvement.

Palmoplantar pustulosis is considered one of the symptoms of SAPHO syndrome. It is a chronic, recurrent dermatological condition characterized by pustules, erythema, dryness, and desquamation on the palms and soles, often accompanied by local discomfort such as itching and pain. The etiology is related to immune factors and smoking. PPP is considered a variant of psoriasis but differs from it, with unclear pathogenesis. Treatment typically includes local and systemic therapies, targeting the underlying cause. About 90% of SAPHO syndrome patients have PPP (24).

Psoriasis vulgaris (PP) is a chronic inflammatory skin disease, an autoimmune disorder characterized by abnormal immune system activity leading to skin cell proliferation, resulting in red, scaly patches with itching, commonly found on elbows, knees, scalp, and lower back. Approximately 14% of SAPHO patients have psoriasis vulgaris (26). Around 3% of psoriatic arthritis (PsA) patients also have SAPHO syndrome (26). Thus, SAPHO was once considered a subtype of PsA.

Acne is a common chronic inflammatory skin disease affecting areas with high sebaceous gland distribution, such as the face, chest, and back. Severe acne manifests as cysts, nodules, and suppurative lesions with redness, pain, and scarring. About 15% of SAPHO syndrome patients have severe acne (26).

Bone-joint involvement is another major symptom of SAPHO syndrome, characterized by osteitis, bone proliferation, and synovitis. Most patients exhibit joint involvement, primarily in the sternoclavicular and spinal joints, manifesting as inflammatory arthritis. About 65%–90% of patients have anterior chest wall involvement (2), typically presenting with erythema, swelling, tenderness, and thickened bone in the affected area. Bone scans show increased radioisotope uptake in the sternoclavicular joints, presenting the “bull’s head sign,” a characteristic feature of SAPHO. About 50% of patients have axial bone involvement, with thoracolumbar spine involvement being the most common (27). Mild bone-joint involvement presents with slight joint swelling and pain, while severe cases may have joint swelling, stiffness, restricted movement, and impact daily life.

Currently, drug-based symptomatic treatment can improve most patients’ skin and bone-joint symptoms to some extent. However, side effects of long-term drug use, such as gastrointestinal ulcers, bleeding, perforation, neurological reactions, and hematopoietic dysfunction, limit prolonged use. When medications are reduced or discontinued, symptoms often recur or worsen, preventing complete symptom relief (24, 28). Studies show that TNF drugs like infliximab can rapidly and effectively alleviate symptoms, especially bone-joint symptoms (29). However, 42.9% of patients with PPP do not improve (15), and some even experience PPP recurrence/worsening or new skin lesions such as psoriasis (30), allergic urticaria (30), psoriasiform skin lesions (31), and fungal skin infections (32). Some patients develop complications such as bronchospasm (33), pneumonia (25), and hair loss (34). Ben Abdelghani et al. (30) reported that four patients showed improvement, but this was temporary, with two experiencing recurrent chest wall pain. Mateo et al. reported that some patients had improved bone-joint symptoms but no improvement or worsening of skin symptoms (35, 36), and some developed new skin lesions (34). One patient developed new bone lesions 10 months after receiving infliximab (37). Some patients experienced worsening primary palmoplantar pustulosis after receiving etanercept, with some developing paradoxical psoriasiform skin lesions or new skin lesions on the trunk and limbs (38). Adalimumab treatment resulted in mild pain relief but was ineffective for osteitis in one patient (39), with PPP recurrence or worsening in others (40), and some even developing disseminated pulmonary tuberculosis (41). IL-1 was effective in only 28.6% of patients (15), while IL-6 was effective in chronic recurrent multifocal osteomyelitis (CRMO) but did not significantly improve skin symptoms (42). The efficacy of IL-23/IL-17 inhibitors in SAPHO is still undetermined. Adamo et al. reported that PDE-4 inhibitors led to worsening joint symptoms (43). Overall, drug treatment side effects cannot be ignored, and clinicians should develop personalized treatment plans based on individual patient conditions to alleviate bone-joint and skin symptoms.

Apart from drug therapy, recent studies suggest that tonsillectomy may become a candidate treatment for SAPHO syndrome. Our summary of these case reports indicates that although tonsillectomy is only suitable for SAPHO patients with recurrent tonsillitis, it shows promising results in these specific patients. Follow-up times ranged from 1 week to 2 years post-tonsillectomy. These patients had varying degrees of skin lesions and bone pain, accompanied by recurrent chronic tonsillitis. Some patients’ SAPHO symptoms worsened due to tonsillitis. Prior to tonsillectomy, patients used first- and second-line drugs or a combination of both to control their condition. About 8% of patients still needed medication post-surgery, with reduced dosages compared to pre-surgery. Short-term follow-ups showed that 25% of patients had worse symptoms than pre-surgery, while long-term follow-ups showed that about 96% of patients experienced varying degrees of skin lesion and bone pain relief. Except for three patients whose recovery was described verbally, the remaining patients’ follow-up data were presented through imaging, pain scales, or lesion area and severity indices. Fifty-six patients’ follow-up data were assessed using various pain scales and lesion scales, including the Visual Analog Scale (VAS), Revised Short Form McGill Pain Questionnaire (SF-MPQ-2), Pain Disability Assessment Scale (PDAS), Palmoplantar Pustulosis Lesion Area and Severity Index (PPPASI). The efficacy of tonsillectomy in SAPHO patients is summarized in Table 1.

In 2020, Wang et al. (18) followed 44 SAPHO patients with chronic tonsillitis who underwent bilateral tonsillectomy. They recorded pre- and postoperative (1, 3, 6, 9, 12, > 12, > 24, > 48 months) skin rash and bone pain using VAS and PPPASI. To better observe the efficacy of tonsillectomy alone, patients stopped using drugs post-surgery. One month post-surgery, 10% and 25% of patients experienced complete resolution of skin lesions and bone pain, respectively. By one year, these rates reached 60% and 80%, respectively. With extended follow-up, these rates continued to rise. At 2 years, three patients experienced 100% resolution of skin lesions and bone pain. However, the sample size was small, and further studies with larger samples are needed to confirm these findings.

Recently, Ma et al. (21) described a 30-year-old female SAPHO patient with a 4-year history of right mandibular bone pain and 1-year history of PPP. Post-tonsillectomy, her bone pain and PPP completely resolved. Xiang et al. (17) published a retrospective study in 2020 showing that tonsillectomy effectively reduced the severity of skin lesions in SAPHO patients. The study included 58 SAPHO patients, with 67.2% suffering from chronic tonsillitis. Ten percent of SAPHO patients experienced worsening skin or bone-joint symptoms due to tonsillitis flare-ups. Seven patients underwent tonsillectomy due to recurrent tonsillitis and worsening SAPHO symptoms post-tonsillitis. Patients were evaluated pre- and post-surgery using pain scales (VAS), PPP lesion area and severity index (PPPASI), and nail psoriasis severity index (NAPSI). Three months post-surgery, patients experienced significant reductions in bone pain and skin lesions, with VAS, PPPASI, and NAPSI scores dropping from 5, 16.2, and 10 to 3, 1.8, and 2, respectively. There were no surgical complications or disease recurrence. Pre-surgery, these seven patients used NSAIDs (etoricoxib) and DMARDs (etanercept, methotrexate, leflunomide) to control their condition. Post-surgery, two patients still required etanercept, but the dosage decreased from 50 mg weekly to 25 mg weekly. Other patients no longer needed medication.

Horiguchi et al. (23) reported a 75-year-old female SAPHO patient with multiple bone-joint pains (wrists, shoulders, upper limbs, and ankles) affecting daily life. Celecoxib, methotrexate, or adalimumab were ineffective in controlling symptoms. Post-bilateral tonsillectomy, her skin and bone pain improved. Quantitative assessments of SAPHO bone pain changes post-tonsillectomy, including VAS, SF-MPQ-2, and PDAS, showed that pre-surgery, the patient’s VAS, SF-MPQ-2, and PDAS scores were 30, 37, and 28, respectively. Three months post-surgery, methotrexate dosage decreased from 8 mg weekly to 6 mg weekly, and skin symptoms significantly improved (presurgery PPPASI was around 1, post-surgery PPPASI was around 0), with VAS, SF-MPQ-2, and PDAS scores dropping to 20, 7, and 19, respectively. Six months post-surgery, the patient no longer needed celecoxib, with VAS, SF-MPQ-2, and PDAS scores of 10, 4, and 24, respectively. Twelve months post-surgery, these scores further improved to 5, 5, and 12, respectively.

Wang et al. (22) reported a 29-year-old female SAPHO patient with lumbar and sacroiliac joint pain and PPP, along with psoriasis on the buttocks and thighs. She experienced severe acute gingivitis (2–3 times per week) at age 21–24, followed by chronic sore throat. Physical examination revealed grade I enlarged tonsils bilaterally with purulent secretions. Non-steroidal anti inflammatory drugs, minocycline, corticosteroids, and Tripterygium wilfordii were ineffective in relieving skin and bone pain. Due to chronic pharyngitis and chronic tonsillitis, she underwent bilateral tonsillectomy. Initially, PPP worsened in the first 2 weeks post-surgery, but improved thereafter. By 6 months post-surgery, PPP completely resolved. Two years later, pharyngeal symptoms disappeared, though bone pain and psoriasis were not reported.

Some SAPHO patients also experience other systemic lesions apart from skin and bone-joint issues. One 53-year-old female patient, in addition to PPP and bilateral sternoclavicular, scapular, and humeral head pain, also had ear pain and fluctuating hearing loss (20). Due to joint pain and PPP, she received steroid treatment, which alleviated rash and bone pain, but hearing loss worsened upon steroid tapering, with air conduction thresholds reaching 53.7 dB. Cyclosporine improved air conduction thresholds to 26.2 dB. Due to PPP exacerbation triggered by tonsil provocation tests, she underwent tonsillectomy, which alleviated PPP and ear pain. Seven months post-surgery, cyclosporine and methotrexate were reduced to control symptoms, but mixed hearing loss did not improve, suggesting irreversible changes in the ossicles.

One 43-year-old female SAPHO patient had hypertrophic pachymeningitis, treated with intravenous methylprednisolone, oral prednisone, and minocycline, which relieved headaches. However, finger joint pain due to PPP still affected daily life. Nine months post-bilateral tonsillectomy, skin, joint symptoms, and headaches almost completely resolved, with reduced prednisone dosage (19). Ten months post-surgery, MRI showed reduced thickening of the dura mater.

The last two case reports are from China, involving a 46-year-old female (44) patient and a 40 years-old male patient (45). Among them, the male patient’s clinical symptoms showed continuous relief within one month after the operation.

In these studies, reports of tonsillectomy complications and SAPHO patient skin and bone pain recurrence are rare, with most patients experiencing short-term improvements and long-term benefits.

## 5 Discussion

Synovitis, acne, pustulosis, hyperostosis, and osteitis (SAPHO) syndrome is a rare autoimmune inflammatory disease characterized by skin and bone-joint lesions, primarily affecting women aged 30–50, with a male-to-female ratio of 1:4. Diagnosis relies on Kahn’s criteria (46). The pathogenesis of SAPHO syndrome is unclear, but immune dysregulation, genetic susceptibility, and environmental factors are widely accepted as contributing factors (5–7). Currently, there is no single treatment that fully addresses all SAPHO-related symptoms, and most treatment data come from case reports, lacking randomized clinical trials.

Tonsil-focused disease (TFD), also known as tonsil focal infection, refers to diseases where tonsillitis causes structural or functional damage to organs distant from the tonsils. Scholars categorize these diseases as “tonsil-induced autoimmune/inflammatory syndromes (TIAS)” which occur due to the immune system’s failure to properly adapt to natural bacteria within the tonsils (47, 48). Tonsillectomy often improves symptoms. Tonsillitis is associated with oral diseases, rheumatoid arthritis, etc., Over the past decade, research has focused on the role of tonsils in diseases caused by bacterial infections or toxin dissemination, such as rheumatic fever, glomerulonephritis, endocarditis, and myocarditis. Recent studies indicate that tonsillectomy effectively treats conditions like PPP (34), pustulotic arthro-osteitis (16), IgA nephropathy (49–51), psoriasis, and reactive arthritis, suggesting associations with tonsillitis.

Due to impaired immune tolerance, PPP patients’ tonsillar mononuclear cells (TMC) exhibit hyperimmune responses to intrinsic bacterial antigens (e.g., α-hemolytic streptococci, Haemophilus parainfluenzae, and/or microbial DNA) (52–56). Abnormal secondary stimulatory molecules in the tonsils lead to T-cell activation and entry into circulation via specific receptors, eventually recruiting to skin lesion areas. T-cells bind to endothelial cells within the skin and migrate through interactions with vascular cell adhesion molecules. Additionally, abnormal immune responses may promote PPP onset by stimulating Th17 responses.

Psoriasis is a chronic immune-mediated skin disease with genetic susceptibility closely linked to immune system dysregulation. Although genetic susceptibility plays a crucial role in psoriasis development, environmental factors like infections trigger and exacerbate the disease. Tonsillitis primarily involves abnormal immune reactions, especially during streptococcal infections, leading to autoimmune responses. This immune response not only triggers skin inflammation through molecular mimicry mechanisms but also exacerbates psoriasis symptoms through excessive cytokine release (57). Streptococcal tonsillitis may trigger abnormal immune responses, including immune complex deposition and molecular mimicry. This can lead to acute glomerulonephritis via renal complex deposition (58) or induce reactive arthritis (59). Studies show that 67.2% of SAPHO patients have tonsillitis, and those with tonsillitis have more severe skin and nail lesions compared to those without tonsillitis. Patients with tonsillitis have significantly higher PPPASI and NAPSI scores (18). In SAPHO patients, skin and bone-joint symptoms significantly improve or disappear even 2 years post-tonsillectomy (18).

The current findings are inherently limited by several methodological constraints. Primarily, the reliance on case reports and small, uncontrolled studies significantly compromises the robustness and internal validity of the conclusions drawn. Furthermore, the absence of quantitative synthesis techniques, such as meta-analysis, precludes a more rigorous assessment of the aggregated evidence presented. The generalizability of these results is also substantially constrained, as the available data are overwhelmingly derived from populations in East Asia, raising questions about their applicability to other ethnic or geographic groups. Finally, a critical gap exists in the form of insufficient long-term follow-up data, which hinders the evaluation of the durability and potential late effects of the observed outcomes. Given the limited evidence and mechanistic plausibility linking tonsillar infection to SAPHO syndrome pathogenesis, there is a compelling need for large-scale, prospective, multicenter studies to rigorously evaluate the efficacy, safety, and long-term outcomes of tonsillectomy as a therapeutic intervention. Such robust research is essential to establish definitive clinical guidelines and address the current evidence gap in managing this complex condition.

## 6 Conclusion

In conclusion, tonsillitis may serve as a local continuous immune stimulus source. Removing this stimulus through tonsillectomy reduces chronic inflammatory responses triggered by tonsillitis, alleviates systemic inflammation, and improves immune dysfunction associated with SAPHO syndrome.

Given the clinical heterogeneity of SAPHO syndrome, individualized treatment is essential. Tonsillectomy in patients with chronic tonsillitis often leads to cessation of medication and resolution of skin and bone pain. A minority of patients still require medication to manage symptoms, but the dosage is reduced compared to pre-surgery. A 2020 study from China reported that 44 SAPHO patients with chronic tonsillitis who underwent bilateral tonsillectomy experienced complete resolution of skin lesions and bone pain in 60% and 80% of cases, respectively, at 12 months post-surgery. Three patients followed for over 2 years showed 100% resolution of skin lesions and bone pain (28). These patients maintained symptom improvement even after stopping SAPHO medications, indicating that tonsillectomy might offer a curative solution without longterm drug dependency. Although tonsillectomy effectively controls symptoms within 2 years, long-term efficacy and recurrence risks require further long-term follow-up studies and larger patient observations. More randomized controlled clinical trials and large-scale studies are needed to accurately assess efficacy and mechanisms. Combining existing immunological research to explore more effective treatments may uncover new targeted therapies to improve SAPHO syndrome treatment outcomes.

## References

[1] Chamot A Vion B Gerster J. Acute pseudoseptic arthritis and palmoplantar pustulosis. *Clin Rheumatol.* (1986) 5:118–23. 10.1007/bf02030980 3514079

[2] Nguyen M Borchers A Selmi C Naguwa S Cheema G Gershwin M. The SAPHO syndrome. *Seminars Arthritis Rheumatism.* (2012) 42:254–65. 10.1016/j.semarthrit.2012.05.006 23153960

[3] Carneiro S Sampaio-Barros PD. SAPHO syndrome. *Rheumatic Dis Clin.* (2013) 39:401–18. 10.1016/j.rdc.2013.02.009 23597971

[4] Przepiera-Będzak H Brzosko M. SAPHO syndrome: pathogenesis, clinical presentation, imaging, comorbidities and treatment: a review. *Adv Dermatol Allergol.* (2021) 38:937–42. 10.5114/ada.2020.97394 35125997 PMC8802951

[5] Liu S Tang M Cao Y Li C. Synovitis, acne, pustulosis, hyperostosis, and osteitis syndrome: review and update. *Therapeutic Adv Musculoskeletal Dis.* (2020) 12:1759720X20912865. 10.1177/1759720x20912865 32523634 PMC7236399

[6] Hedrich CM Morbach H Reiser C Girschick HJ. New insights into adult and paediatric chronic non-bacterial osteomyelitis CNO. *Curr Rheumatol Rep.* (2020) 22:1–11. 10.1007/s11926-020-00928-1 32705386 PMC7378119

[7] Berthelot JM Corvec S Hayem G. SAPHO, autophagy, IL-1, FoxO1, and Propionibacterium (Cutibacterium) acnes. *Joint Bone Spine.* (2018) 85:171–6. 10.1016/j.jbspin.2017.04.010 28499891

[8] Li Y Huo J Cao Y Yu M Zhang Y Li Z Efficacy of tofacitinib in synovitis, acne, pustulosis, hyperostosis and osteitis syndrome: a pilot study with clinical and MRI evaluation. *Ann Rheumatic Dis.* (2020) 79:1255–7. 10.1136/annrheumdis-2020-217250 32332076

[9] Yang Q Zhao Y Li C Luo Y Hao W Zhang W. Case report: successful treatment of refractory SAPHO syndrome with the JAK inhibitor tofacitinib. *Medicine.* (2018) 97:e11149. 10.1097/md.0000000000011149 29924019 PMC6024477

[10] Wenwen S Guangjin W. Refractory SAPHO syndrome treated with tofacitinib: a case report. *China J Leprosy Skin Dis.* (2024) 40:50–2. 10.12144/zgmfskin202401050

[11] Baisya R Gavali M Tyagi M Devarasetti PK. A case of SAPHO syndrome complicated by uveitis with good response to both TNF inhibitor and JAKinib. *Case Rep Rheumatol.* (2023) 2023:6201887. 10.1155/2023/6201887 36712597 PMC9876693

[12] Li B Li GW Xue L Chen YY. Rapid remission of refractory synovitis, acne, pustulosis, hyperostosis, and osteitis syndrome in response to the Janus kinase inhibitor tofacitinib: a case report. *World J Clin Cases.* (2020) 8:4527. 10.12998/wjcc.v8.i19.4527 33083414 PMC7559655

[13] Xin P Xu X Deng C Liu S Wang Y Zhou X The role of JAK/STAT signaling pathway and its inhibitors in diseases. *Int Immunopharmacol.* (2020) 80:106210. 10.1201/9781351042468-1 31972425

[14] Ucciferri C Vecchiet J Falasca K. Role of monoclonal antibody drugs in the treatment of COVID-19. *World J Clin Cases.* (2020) 8:4280. 10.12998/wjcc.v8.i19.4280 33083387 PMC7559676

[15] Cheng W Li F Tian J Xie X Chen JW Peng XF New insights in the treatment of SAPHO syndrome and medication recommendations. *J Inflammation Res.* (2022) 15:2365–80. 10.2147/jir.s353539 35444448 PMC9013916

[16] Takahara M Hirata Y Nagato T Kishibe K Katada A Hayashix T Treatment outcome and prognostic factors of tonsillectomy for palmoplantar pustulosis and pustulotic arthro-osteitis: a retrospective subjective and objective quantitative analysis of 138 patients. *J Dermatol.* (2018) 45:812–23. 10.1111/1346-8138.14348 29732605

[17] Xiang Y Wang Y Cao Y Li Z Xiong D Wang L Tonsillitis as a possible predisposition to synovitis, acne, pustulosis, hyperostosis and osteitis (SAPHO) syndrome. *Int J Rheumatic Dis.* (2021) 24:519–25. 10.1111/1756-185x.14064 33502120

[18] Wang Y Yirong X Chen L Wen Z Zhiqiang G. Improvement of bone pain and rash symptoms in SAPHO patients after tonsillectomy. *Basic Med Clin Pract.* (2020) 40:1671–6. 10.16352/j.issn.1001-6325.2020.12.016

[19] Shiraishi W Hayashi S Iwanaga Y Murai H Yamamoto A Kira JI. A case of synovitis, acne, pustulosis, hyperostosis, and osteitis (SAPHO) syndrome presenting with hypertrophic pachymeningitis. *J Neurol Sci.* (2015) 349:229–31. 10.1016/j.jns.2014.12.020 25549534

[20] Shimizu S Yukawa K Kawaguchi S Okubo Y Suzuki M. Fluctuating mixed-type hearing loss associated with synovitis–acne–pustulosis–hyperostosis–osteomyelitis (SAPHO) syndrome. *Auris Nasus Larynx.* (2010) 37:508–10. 10.1016/j.anl.2009.09.008 19864094

[21] Ma M Wu X Cao Y Liu Y Liu S Li C. Synovitis, acne, pustulosis, hyperostosis, osteitis (SAPHO) syndrome associated with pyoderma gangrenosum: report of two patients. *Int J Dermatol.* (2023) 62:e186–9. 10.1111/ijd.16492 36345566

[22] Wang Y Xiang Y Cao Y Zhang W Li C. Tonsillectomy leads to remission of bone marrow edema and palmoplantar pustulosis in synovitis, acne, pustulosis, hyperostosis, and osteitis syndrome. *J Clin Rheumatol.* (2021) 27:S719–20. 10.1097/rhu.0000000000001546 32897988

[23] Horiguchi S Fujita T Kinoshita K Doi K. Tonsillectomy as an effective treatment for arthralgia of SAPHO syndrome. *J Surg Case Rep.* (2020) 2020:rjaa288. 10.1093/jscr/rjaa288 32934788 PMC7479647

[24] Hayem G Bouchaud-Chabot A Benali K Roux S Palazzo E Silbermann-Hoffman O SAPHO syndrome: a long-term follow-up study of 120 cases. *Semin Arthritis Rheumatism.* (1999) 29:159–71. 10.1016/s0049-0172(99)80027-4 10622680

[25] Grosjean C Hurtado-Nedelec M Nicaise-Roland P Ferreyra-Dillon R Bollet C Quintin E Prevalence of autoantibodies in SAPHO syndrome: a single-center study of 90 patients. *J Rheumatol.* (2010) 37:639–43. 10.3899/jrheum.090863 20110527

[26] Demirci Yildirim T Sari İ. SAPHO syndrome: current clinical, diagnostic and treatment approaches. *Rheumatol Int.* (2024) 44:2301–13. 10.1007/s00296-023-05491-3 37889264

[27] Li C Ye Y Cao Y Zhang W Xu W Wu N Axial skeletal lesions and disease duration in SAPHO syndrome: A retrospective review of computed tomography findings in 81 patients. *Int J Rheumatic Dis.* (2020) 23:1152–8. 10.1111/1756-185x.13899 32588963

[28] Hayama K Inadomi T Fujisawa D Terui T. A pilot study of medium-dose cyclosporine for the treatment of palmoplantar pustulosis complicated with pustulotic arthroosteitis. *Eur J Dermatol.* (2010) 20:758–62. 10.1684/ejd.2010.1109 21047721

[29] Daoussis D Konstantopoulou G Kraniotis P Sakkas L Liossis SN. Biologics in SAPHO syndrome: a systematic review. *Semin Arthritis Rheumatism.* (2019) 48:618–25. 10.1016/j.semarthrit.2018.04.003. 29773231

[30] Abdelghani KB Dran DG Gottenberg JE Morel J Sibilia J Combe B. Tumor necrosis factor-α blockers in SAPHO syndrome. *J Rheumatol.* (2010) 37:1699–704. 10.3899/jrheum.091086 20472920

[31] Anić B Padjen I Barešić M Težak S. The lobster sign in SAPHO syndrome: unusually extensive osteitis of the anterior chest wall partially responsive to infliximab. *Rheumatol Int.* (2014) 34:281–2. 10.1007/s00296-012-2606-y 23263495

[32] Eleftheriou D Gerschman T Sebire N Woo P Pilkington CA Brogan PA. Biologic therapy in refractory chronic non-bacterial osteomyelitis of childhood. *Rheumatology.* (2010) 49:1505–12. 10.1093/rheumatology/keq122 20430869

[33] Wagner AD Andresen J Jendro MC Hülsemann JL Zeidler H. Sustained response to tumor necrosis factor α–blocking agents in two patients with SAPHO syndrome. *Arthritis Rheumatism.* (2002) 46:1965–8. 10.1002/art.10539 12124882

[34] Li C Wu X Cao Y Zeng Y Zhang W Zhang S Paradoxical skin lesions induced by anti-TNF-α agents in SAPHO syndrome. *Clin Rheumatol.* (2019) 38:53–61. 10.1007/s10067-018-4083-5 29611085

[35] Mateo L Sanint J Muguruza SR Morillo MM Andrés RP Puigcerver SD. SAPHO syndrome presenting as an osteolytic lesion of the neck. *Reumatol Clín.* (2017) 13:44–7. 10.1016/j.reumae.2015.11.022 26793990

[36] Massara A Cavazzini PL Trotta F. In SAPHO syndrome anti-TNF-α therapy may induce persistent amelioration of osteoarticular complaints, but may exacerbate cutaneous manifestations. *Rheumatology.* (2006) 45:730–3. 10.1093/rheumatology/kei221 16403830

[37] Fruehauf J Cierny-Modrè B Caelen LE Schwarz T Weinke R Aberer E. Response to infliximab in SAPHO syndrome. *Case Rep.* (2009) 2009:bcr1020081145. 10.1136/bcr.10.2008.1145 21686446 PMC3029502

[38] Zhang X Wu X Li C. Successful treatment of synovitis, acne, pustulosis, hyperostosis, and osteitis and paradoxical skin lesions by Tripterygium wilfordii hook f: a case report. *J Int Med Res.* (2020) 48:0300060520949100. 10.1177/0300060520949100 32962502 PMC7518000

[39] Henriques CC Sousa M Panarra A Riso N. The dark side of SAPHO syndrome. *Case Rep.* (2011) 2011:bcr1120115197. 10.1136/bcr.11.2011.5197 22670011 PMC3246173

[40] Cianci F Zoli A Gremese E Ferraccioli G. Clinical heterogeneity of SAPHO syndrome: challenging diagnose and treatment. *Clin Rheumatol.* (2017) 36:2151–8. 10.1007/s10067-017-3751-1 28725947

[41] Hess S Hospach T Nossal R Dannecker G Magdorf K Uhlemann F. Lifethreatening disseminated tuberculosis as a complication of TNF-α blockade in an adolescent. *Eur J Pediatrics.* (2011) 170:1337–42. 10.1007/s00431-011-1501-y 21625932

[42] Fujita S Kosaka N Mito T Hayashi H Morita Y. Development of aseptic subcutaneous abscess after tocilizumab therapy in a patient with SAPHO syndrome complicated by amyloid A amyloidosis. *Int J Rheumatic Dis.* (2015) 18:476–9. 10.1111/1756-185x.12525 25545988

[43] Adamo S Nilsson J Krebs A Steiner U Cozzio A French LE Successful treatment of SAPHO syndrome with apremilast. *Br J Dermatol.* (2018) 179:959–62. 10.1111/bjd.16071 29034454

[44] Jia H Li Y Liu Z Guo Y. SAPHO syndrome with chronic tonsillitis: a case report and literature review. *Lin Chuang er bi yan hou tou Jing wai ke za zhi.* (2021) 35:944–7. 10.13201/j.issn.2096-7993.2021.10.018 34628822 PMC10127713

[45] Xie YF Zeng J Liu ZQ. Tonsillectomy as a treatment for SAPHO syndrome: a case report. *Zhonghua Er Bi Yan Hou Tou Jing Wai Ke Za Zhi.* (2021) 56:1102–4. 10.3760/cma.j.cn115330-20201217-00929 34666473

[46] Kahn MF Khan MA. The SAPHO syndrome. *Bailliere’s Clin Rheumatol.* (1994) 8:333–62. 10.1016/s0950-3579(94)80022-7 8076391

[47] Harabuchi Y Takahara M. Recent advances in the immunological understanding of association between tonsil and immunoglobulin a nephropathy as a tonsil-induced autoimmune/inflammatory syndrome. *Immunity Inflammation Dis.* (2019) 7:86–92. 10.1002/iid3.248 30957421 PMC6485698

[48] Harabuchi Y Takahara M. Pathogenic role of palatine tonsils in palmoplantar pustulosis: a review. *J Dermatol.* (2019) 46:931–9. 10.1111/1346-8138.15100 31556151

[49] Hotta O Miyazaki M Furuta T Tomioka S Chiba S Horigome I Tonsillectomy and steroid pulse therapy significantly impact on clinical remission in patients with IgA nephropathy. *Am J Kidney Dis.* (2001) 38:736–43. 10.1080/03655230410003387 11576876

[50] Kawamura T Yoshimura M Miyazaki Y Okamoto H Kimura K Hirano K A multicenter randomized controlled trial of tonsillectomy combined with steroid pulse therapy in patients with immunoglobulin A nephropathy. *Nephrol Dialysis Transpl.* (2014) 29:1546–53. 10.1093/ndt/gfu020 24596084 PMC4106640

[51] Komatsu H Sato Y Miyamoto T Tamura M Nakata T Tomo T Significance of tonsillectomy combined with steroid pulse therapy for IgA nephropathy with mild proteinuria. *Clin Exp Nephrol.* (2016) 20:94–102. 10.1007/s10157-015-1138-7 26123429 PMC4756031

[52] Murakata H Harabuchi Y Kataura A. Increased Interleukin-6, interferon-? and tumour necrosis Factor-a production by tonsillar mononuclear cells stimulated with alpha-streptococci in patients with pustulosis palmaris et plantaris. *Acta Otolaryngol.* (1999) 119:384–91. 10.1080/00016489950181431 10380747

[53] Kukuminato Y Shido F. Investigation of the immune response in tonsillar lymphocytes against streptococci in patients with pustulosis palmaris et plantaris. *Nippon Jibiinkoka Gakkai Kaiho.* (1990) 93:949–61. 10.3950/jibiinkoka.93.949 2213357

[54] Kukuminato Y Shido F. Investigation of the bacterial flora in the tonsillar lacunae and serum levels of streptococcal antigen-specific antibodies in patients with pustulosis palmaris et plantaris. *Nippon Jibiinkoka Gakkai Kaiho.* (1990) 93:786–95. 10.3950/jibiinkoka.93.786 2384834

[55] Murakata H Harabuchi Y Kukuminato Y Yokoyama Y Kataura A. Cytokine production by tonsillar lymphocytes stimulated with alpha-streptococci in patients with pustulosis palmaris et plantaris. *Acta oto-laryngologica. Supplementum.* (1996) 523:201–3. 9082782

[56] Sigurdardottir S Thorleifsdottir R Valdimarsson H Johnston A. The role of the palatine tonsils in the pathogenesis and treatment of psoriasis. *Br J Dermatol.* (2013) 168:237–42. 10.1111/j.1365-2133.2012.11215.x 22901242

[57] Loeb HW. Acute nephritis following acute tonsillitis. *J Am Med Assoc.* (1910) 55:1705–8. 10.1001/jama.1910.04330200015004

[58] Nissen HA. Arthritis and tonsillar infection. *N Engl J Med.* (1935) 212:1027–33. 10.1056/nejm193505302122203

[59] Brandes M Willimann K Moser B. Professional antigen-presentation function by human γδ T cells. *Science.* (2005) 309:264–8. 10.1126/science.1110267 15933162

